# Temporal Trends in Upper Gastrointestinal Endoscopy: A Comprehensive Audit Comparing the Pre-COVID, COVID, and Post-COVID Eras for Quality Enhancement

**DOI:** 10.7759/cureus.80137

**Published:** 2025-03-06

**Authors:** Vinayak Venu, Khadeija Hussain, Balamurugan G., Abhinav C. G., Ajay H Bhandarwar, Nethra R Jain

**Affiliations:** 1 General Surgery, Grant Government Medical College and Sir Jamshedjee Jeejeebhoy (JJ) Group of Hospitals, Mumbai, IND; 2 General Surgery, Princess Royal University Hospital, London, GBR

**Keywords:** covid-19, gastrointestinal endoscopy, surgical gastro, time trends, ugi scopy

## Abstract

Upper gastrointestinal (UGI) endoscopy, vital for diagnosing and managing gastrointestinal diseases, experienced challenges in continuity and training during the COVID-19 pandemic. This study evaluates trends in UGI endoscopy procedures across pre-COVID, COVID, and post-COVID periods at a government tertiary care institute, assessing volumes, common indications and findings, trainee involvement, and service delivery barriers. This retrospective study reviewed 2,165 patient records from January 2018 to February 2023, including demographics, procedural details, indications, findings, and trainee participation. Descriptive statistics were used to analyze trends and service interruptions caused by equipment failures. Despite the challenges posed by the pandemic, proactive equipment maintenance and crisis response remain essential. Enhancing endoscopy training programs is crucial for maintaining service quality and continuity during healthcare crises. Continuous improvement efforts are important for optimizing patient care and mitigating future disruptions.

## Introduction

Upper gastrointestinal (UGI) endoscopy, or oesophago-gastro-duodenoscopy (OGD), is the gold standard diagnostic modality for UGI diseases [1]. Its significance lies in its ability to serve both diagnostic and therapeutic purposes, now made possible by the advent of novel therapeutic techniques and equipment [2]. It is worth noting that many benign UGI diseases necessitate a preliminary diagnostic UGI endoscopy to make appropriate management decisions [3].

In a tertiary care government setting, diagnostic UGI endoscopy provides invaluable services to patients who are unable to afford private alternatives. This accessibility consequently attracts a larger patient population. However, disruptions to these services due to equipment failures and maintenance issues result in detrimental consequences, such as a high volume of patient referrals elsewhere and significant adversities for both patients and their families.

The COVID-19 pandemic has further compounded the challenges in maintaining uninterrupted endoscopy services. As a result, a full recovery in service provision has not yet been achieved. Furthermore, the substantial volume of UGI endoscopies presents valuable learning opportunities for trainees seeking to develop their endoscopic skills. Thus, our audit aimed to evaluate the number of endoscopic procedures conducted over five years, identify barriers to maintaining continuous endoscopy services, and examine training opportunities available for trainees in this area.

## Materials and methods

Study design

This study was a comprehensive retrospective observational analysis focusing on patients who underwent diagnostic UGI endoscopy at a government tertiary care teaching hospital. The period covered was from January 2018 to February 2023, providing a unique perspective that spans the pre-pandemic, pandemic, and post-pandemic phases of COVID-19. By examining this extended timeframe, the study could assess changes in endoscopy volume and practice patterns, offering insights into the pandemic's effects on both patient care and medical training. The study period enabled a robust comparison of how diagnostic gastrointestinal endoscopy services evolved during times of healthcare system strain and how recovery and adaptations were made post-pandemic.

Objectives

Primary Objective

The primary objective of the study was to assess the volume of diagnostic UGI endoscopies performed during the pre-COVID, COVID-19, and post-COVID periods, providing a clear comparison across these critical phases.

Secondary Objectives

The secondary objectives included: (i) to explore the availability and quality of training opportunities for medical trainees during these periods; (ii) to identify the challenges encountered in maintaining endoscopy services, particularly during the pandemic; and (iii) to investigate the common UGI symptoms observed during endoscopy and correlate them with the corresponding endoscopic findings.

Patient recruitment and procedure

Patients scheduled for diagnostic UGI endoscopy were instructed to fast from midnight prior to the procedure. The endoscopic procedures were carried out in a dedicated endoscopy suite, with local anesthesia administered via a 2% lignocaine spray for patient comfort. Procedures were routinely performed in the morning, during standard working hours, ensuring consistency in the timing of interventions.

Prior to the procedure, patient-reported symptoms were documented and informed written consent was obtained. After each endoscopy, the findings were communicated to the patient, and they were provided with a detailed report. Post-procedure, appropriate prescriptions and care instructions were given based on the findings.

Participant inclusion and exclusion

Inclusion Criteria

The study included both outpatient and elective inpatient cases where diagnostic endoscopy was indicated. This ensured that a broad spectrum of cases across various clinical settings was captured. *Exclusion Criteria*

Due to limited access to therapeutic endoscopy equipment and the necessary expertise, emergency cases were referred to specialty centers and were not included in the study.

Data collection

Data were extracted from the operating room (OR) registers, ensuring accuracy and completeness. The information collected included patient demographics (age and gender), reported symptoms before the procedure, endoscopic findings (e.g., ulcers, gastritis, and tumors), the date of the procedure, patient category (outpatient or inpatient), and the level of personnel performing the procedure (trainee, junior consultant, senior consultant, or professor).

Data management and analysis

All data were organized and recorded in a Microsoft Excel 2019 spreadsheet (Microsoft Corp., Redmond, US) for initial management. Statistical analysis was then conducted using IBM SPSS Statistics version 29 (IBM Corp., Armonk, US). Descriptive statistics were employed to summarize the data: continuous variables (e.g., patient age and procedure volumes) were presented as means with standard deviations, and categorical variables (e.g., gender, patient category, symptoms, and findings) were expressed as percentages.

The analysis aimed to identify trends, challenges, and outcomes related to diagnostic endoscopy across the pre-pandemic, pandemic, and post-pandemic periods, providing valuable insights into the evolution of service delivery and trainee education in the context of a global health crisis.

## Results

Patient demographics

**Figure 1:**
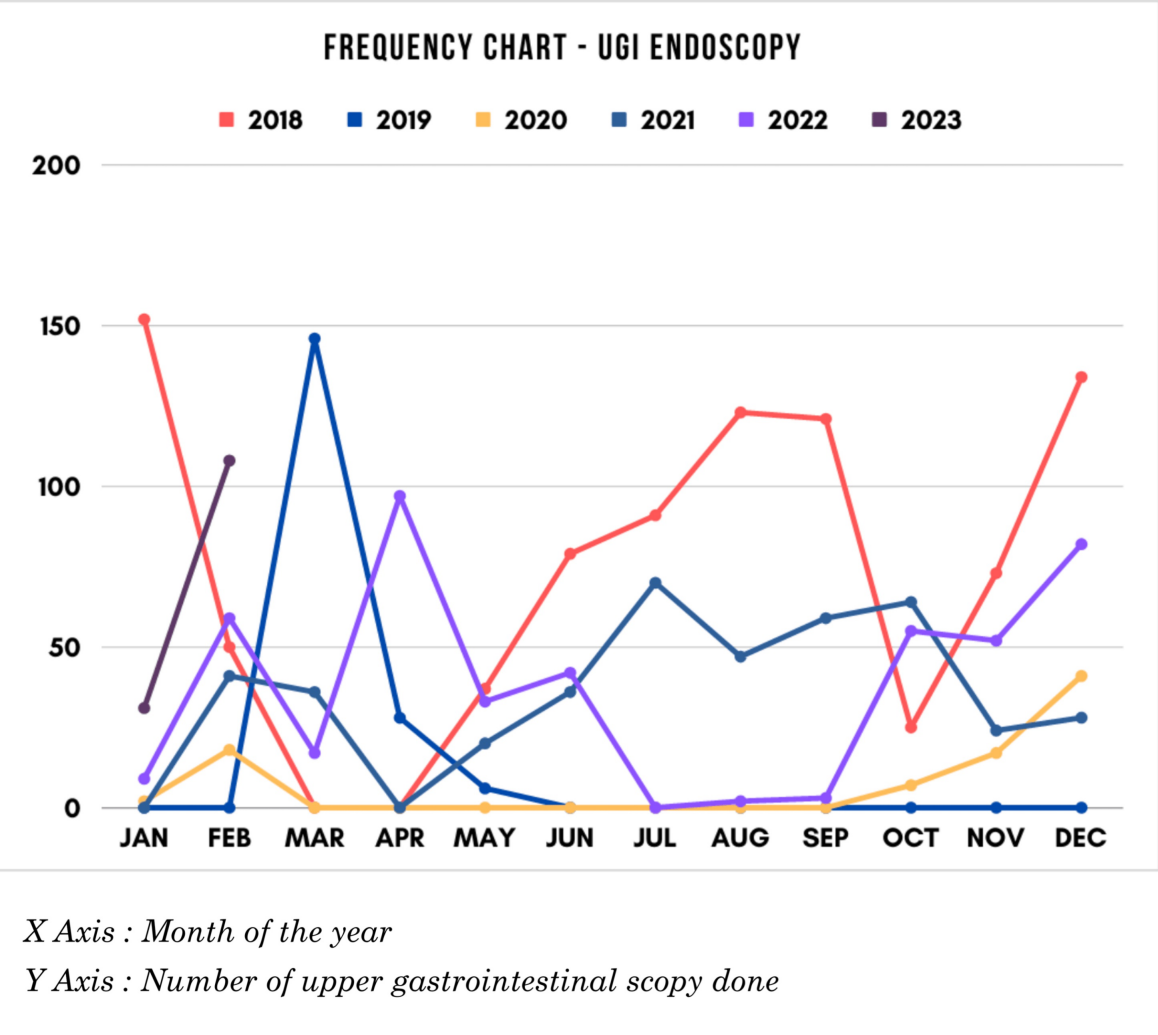
Annual frequency of endoscopy services in our study UGI: Upper gastrointestinal

A retrospective analysis was conducted using 2,165 patient records extracted from the endoscopy register between January 2018 and February 2023 (Figure 1). The mean age of the study population was 42.80 years, with a standard deviation of 15.76 years (Table 1). Among the participants, 60% were male and 40% were female. Outpatients constituted the majority of the study population (1349, 62.3%), while inpatients accounted for the remainder (816, 37.7%).

**Table 1:** Patient demographics

Year	Sample (N)	Age in years (mean ± SD, range)	Male (n, %)	Female (n, %)
2018	885	42.51 ± 15.834 (12-95)	561 (63.4%)	324 (36.6%)
2019	180	45.17 ± 17.266 (17-85)	118 (65.6%)	62 (34.4%)
2020	85	42.54 ± 14.115 (13-80)	57 (67.1%)	28 (32.9%)
2021	425	41.48 ± 14.558 (14-85)	245 (57.6%)	180 (42.4%)
2022	451	43.65 ± 15.867 (13-85)	245 (54.3%)	206 (45.7%)
2023	139	43.01 ± 17.172 (14-95)	76 (54.7%)	63 (45.3%)
2018-2023	2165	42.80 ± 15.764 (12-95)	1302 (60.1%)	863 (39.9%)

Indications of UGI endoscopy

Amongst the indications for diagnostic UGI endoscopy, epigastric pain was found to be the most common (51.2%), followed by dyspepsia (18.9%) and heartburn (14.1%). The remaining indications included regurgitation, vomiting, bloating, dysphagia, hematemesis, anorexia, anaemia, weight loss, chest pain, voice change, foreign body ingestion, and malaena (Table 2).

**Table 2:** Indications for UGI endoscopy UGI: Upper gastrointestinal

Symptom	No. of patients (%)
Epigastric pain	51.2
Dyspepsia	18.9
Heartburn	14.1
Regurgitation	13.2
Vomiting	12.6
Bloating	9.5
Dysphagia	6.1
Hematemesis	3.6
Anorexia	3.6
Anaemia	2.9
Weight loss	2.6
Chest pain	2.1
Voice change	0.6
Foreign body ingestion	0.4
Malaena	0.5

Findings of UGI endoscopy

Of the 2,165 patients, 11.5% had no abnormalities detected during diagnostic UGI endoscopy (Table 3). Amongst those who did have abnormalities, gastritis was the most common finding, with 731 patients (33.8%) having this diagnosis. Hiatus hernia was the second most prevalent finding, identified in 321 patients (14.8%), followed by oesophageal varices in 130 patients (6%).

**Table 3:** UGI endoscopy findings in our study population UGI: Upper gastrointestinal

S.No.	UGI endoscopy findings	No. of patients (n, %)
1	Atrophic gastritis	17 (0.79%)
2	Barret’s oesophagus	3 (0.14%)
3	Candidiasis	22 (1.02%)
4	Corrosive gastritis	3 (0.14%)
5	Corrosive oesophagitis	2 (0.09%)
6	Duodenal diverticulum	1 (0.05%)
7	Duodenal growth/ulcer	8 (0.37%)
8	Duodenal stricture/narrowing	2 (0.09%)
9	Duodenitis	46 (2.12%)
10	Fistula (tracheo-oesophageal/gastro-colic)	3 (0.14%)
11	Foreign body	4 (0.18%)
12	Gastric antral vascular ectasia	2 (0.09%)
13	Gastric growth/ulcer	48 (2.22%)
14	Gastric stricture/narrowing	2 (0.09%)
15	Gastritis	731 (33.8%)
16	Gastritis + duodenitis	79 (3.65%)
17	Hiatus hernia	321 (14.8%)
18	Hiatus hernia + duodenitis	8 (0.37%)
19	Hiatus hernia + gastritis	110 (5.08%)
20	Hiatus hernia + gastritis + duodenitis	8 (0.37%)
21	Hiatus hernia + oesophagitis	19 (0.88%)
22	Hiatus hernia + oesophagitis + gastritis	9 (0.42%)
23	Incomplete study/inadequate preparation	52 (2.40%)
24	Laryngitis	5 (0.23%)
25	Normal study	248 (11.5%)
26	Oesophageal + gastric varices	19 (0.88%)
27	Oesophageal growth/ulcer	35 (1.62%)
28	Oesophageal stricture/narrowing	43 (1.99%)
29	Oesophageal varices	130 (6.00%)
30	Oesophagitis	51 (2.36%)
31	Oesophagitis + gastritis	45 (2.08%)
32	Oesophagitis + gastritis + duodenitis	4 (0.18%)
33	Percutaneous endoscopy gastrostomy tube removal	3 (0.14%)
34	Portal gastropathy	70 (3.23%)
35	Post-cricoid narrowing/growth	12 (0.55%)

Endoscopy training

The procedure was conducted by both junior and senior consultants, as well as trainees who were under the supervision of their respective consultants. Initially, the majority of the procedures were performed by junior and senior consultants. However, the involvement of trainees in performing these procedures gradually increased over time (Table 4). By 2023, trainees accounted for up to 43.88% of the procedures performed.

**Table 4:** Comparison of the number of endoscopies performed by grade throughout the study period

S.No.	Year	Grade	Frequency (n, %)
1	2018	Trainee	10 (1.13%)
		Junior consultant	615 (69.49%)
		Senior consultant	260 (29.38%)
2	2019	Trainee	36 (20%)
		Junior consultant	69 (38.33%)
		Senior consultant	75 (41.67%)
3	2020	Trainee	8 (9.41%)
		Junior consultant	53 (62.35%)
		Senior consultant	24 (28.24%)
4	2021	Trainee	35 (8.24%)
		Junior consultant	212 (49.88%)
		Senior consultant	178 (41.88%)
5	2022	Trainee	52 (11.53%)
		Junior consultant	218 (48.34%)
		Senior consultant	181 (40.13%)
6	2023	Trainee	61 (43.88%)
		Junior consultant	46 (33.09%)
		Senior consultant	32 (23.02%)

Barriers to endoscopy services

**Figure 2:**
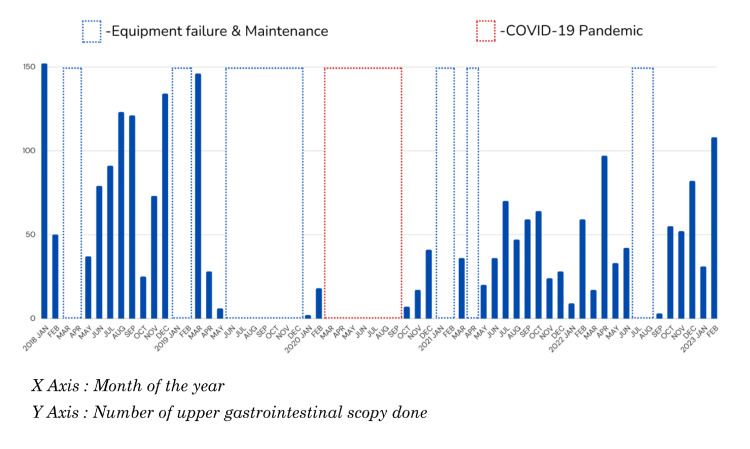
Bar chart representing the temporal trends in monthly endoscopy services across pre-COVID, COVID, and post-COVID eras

Despite the significant impact of the COVID-19 pandemic on endoscopic services, it is noteworthy that only a small portion of the interruption was attributed to its effects. The majority of the service interruptions were due to equipment failures, which proved to be time-consuming in terms of repair and reintegration into the service stream, particularly within the constraints of a government setting (Figure 2). These findings emphasize the importance of expedited processes for equipment repair and maintenance in order to achieve consistent numbers of endoscopy services throughout the year with minimal fluctuations.

## Discussion

UGI symptoms pose a substantial burden on a global scale, with various studies indicating that approximately 20-25% of the population experiences UGI symptoms. While the majority of the symptoms resolve, there exists a subset of patients who experience chronic symptoms, prompting them to seek necessary treatment. Despite initial medical interventions, the symptoms of some patients persist, necessitating further evaluation through UGI endoscopy. The focus of our study was to analyse the patterns and trends of UGI endoscopy services provided within our hospital. The retrospective analysis of 2,165 patients provided valuable insights into the demographics, indications, findings, and other pertinent aspects of UGI endoscopy services.

In light of the results and interpretations presented in this study, several key areas for quality improvement in UGI endoscopy services can be identified. First and foremost, addressing the issue of equipment failures, which accounted for the majority of service interruptions, is paramount. Developing a proactive equipment maintenance team and implementing rapid-response algorithms for repairs are essential steps in ensuring continuous and reliable service. This may involve regular equipment assessments, predictive maintenance models, and investment in updated technology to minimize downtime. A research project conducted in the UK involved the application of Cognitive-Task Analysis (CTA) and Time-Motion Study (TMS) methodologies across three endoscopy units. The primary objective was to comprehensively map out the various conventional tasks associated with endoscopy procedures when unexpected disruptions occur. Ultimately, the study culminated in the development and proposal of a digital reporting tool designed to enhance the efficiency of endoscopy processes [4].

Another critical concern was the profound impact of the COVID-19 pandemic. This global health crisis had a significant and far-reaching effect on our operations. The majority of elective procedures, including diagnostic endoscopic services, had to be either cancelled or rescheduled. Many emergency procedures also faced delays. Our institute's endoscopy service was similarly affected by these disruptions. A multicentre survey conducted in Egypt disclosed that 34.8% of the 39 centres surveyed experienced a shortage of staff. This staffing deficiency was identified as a significant obstacle to resuming services by 86.4% of the centres according to per-protocol analysis [5]. Similarly, a questionnaire-based study conducted in the UK surveyed 97 endoscopic specialists and found that 20% of these services were not provided during the COVID-19 pandemic [6].

Another significant concern revolved around the interruption of endoscopy service training, which was severely impeded. An international survey encompassing 770 trainees from 63 different countries revealed that 93.8% of participants experienced a reduction in their endoscopy case volume. Additionally, a substantial 71.9% of participants expressed concerns that the COVID-19 pandemic might extend the duration of their training. This challenging situation led to anxiety in 52.4% of respondents and burnout in 18.8% [7]. Furthermore, as trainees increasingly contribute to performing UGI endoscopy procedures, maintaining high standards of training and supervision is crucial. Institutions should establish structured training programs with defined competencies, regular evaluations, and mentorship to ensure that trainees achieve proficiency in a safe and effective manner.

In response to the exponential rise in COVID-19 cases across Italy, Spain, and other parts of Europe, and the anticipated surge in cases in the US, the American College of Surgeons called for the minimization, postponement, or cancellation of elective surgeries, endoscopies, and other invasive procedures until the peak of COVID-19 transmission had passed [8]. Additionally, they recommended limiting the use of ICU beds and ensuring the use of maximum personal protective equipment (PPE). The US Surgeon General also advised hospitals to delay elective surgeries.

On March 15, 2020, the American Association for the Study of Liver Diseases, the American College of Gastroenterology, the American Gastroenterological Association, and the American Society of Gastrointestinal Endoscopy (ASGE) issued a joint statement addressing the evolving COVID-19 situation [9]. In this message, the societies urged gastrointestinal physicians to reschedule elective, non-urgent endoscopic procedures, and further recommended classifying procedures into "non-urgent/postpone" and "non-urgent/perform" based on necessity.

The European Society of Gastrointestinal Endoscopy (ESGE) also released a position statement regarding the risk of transmission among healthcare professionals in endoscopy units [10]. According to the ESGE, patients should be risk stratified based on their symptoms, travel history, and contact with known COVID-19 patients [11]. Furthermore, PPE usage should be tailored to the patient's risk level, with high-risk individuals requiring two pairs of gloves, respiratory masks, and other protective equipment. ESGE also recommended contacting patients at least 24 hours prior to the procedure and implementing post-procedure risk management by checking in with patients 1-2 weeks after the procedure to monitor for any symptoms.

In April 2020, national and international societies issued guidance documents on the prioritization of procedures [10,12-14]. These recommendations share similarities in their guidance for urgent and emergent cases, aligning with our triage criteria. Cases falling outside these criteria were evaluated on a case-by-case basis. For instance, elective variceal eradication procedures were performed in patients with recent bleeding to prevent rebleeding episodes, and endoscopic lesion resections (endoscopic mucosal resection (EMR) or endoscopic submucosal dissection (ESD)) were carried out in high-risk patients. Each case of dysphagia was carefully assessed before the procedure, particularly when associated with alarm features. Additionally, some instances of acute gastrointestinal bleeding occurred in patients with a history of inflammatory bowel disease, often necessitating hospitalization due to severe disease activity. These cases were also managed in accordance with international recommendations [15].

A study from a tertiary general university hospital in Greece found that the COVID-19 pandemic led to a significant reduction in emergency surgical operations and hospital admissions due to widespread fear and anxiety. While the total number of acute admissions remained unchanged, patients during the COVID era had longer hospital stays and operation durations, likely due to delayed presentation and more severe clinical conditions [16].

Finally, given the diverse range of findings in UGI endoscopy, a multidisciplinary approach to patient management and follow-up is advisable. Collaboration between gastroenterologists, pathologists, and other specialists can facilitate accurate diagnosis and optimal treatment plans for patients with complex or rare UGI conditions. Incorporating these quality improvement strategies can enhance the overall effectiveness of UGI endoscopy services, ultimately leading to improved patient care and outcomes. Continuous monitoring and adaptation based on data-driven insights will be essential in the pursuit of excellence in gastrointestinal healthcare delivery.

Limitations of the study

The retrospective nature of the study highlights the reliance on previously recorded data, which may result in incomplete symptom documentation and inconsistent endoscopy practices. Additionally, since the findings are based on a single government hospital, they may not be generalizable to other institutions, particularly those with more advanced endoscopy capabilities. Furthermore, the limited access to therapeutic procedures, due to the exclusion of emergency cases and therapeutic interventions, restricts the analysis to diagnostic endoscopy, thereby missing valuable insights on therapeutic procedures conducted during the pandemic.

## Conclusions

In conclusion, our analysis of UGI endoscopy services highlights critical areas for improvement in gastroenterological care. Equipment failure, a recurring yet pertinent issue, disrupts service continuity and requires proactive maintenance programs and technology upgrades to ensure uninterrupted service. The COVID-19 pandemic's impact emphasizes the need for adaptive crisis management, resource allocation, and contingency planning to maintain service excellence during healthcare crises. Disruptions in the learning for trainees emphasize the necessity for structured programs, mentorship, and supportive environments to ensure safe and proficient trainee development.
